# Signature of Pareto optimization in the *Escherichia coli* proteome

**DOI:** 10.1038/s41598-018-27287-3

**Published:** 2018-06-14

**Authors:** Loren Koçillari, Piero Fariselli, Antonio Trovato, Flavio Seno, Amos Maritan

**Affiliations:** 1 INFN and Dipartimento di Fisica e Astronomia ‘G. Galilei’, Università di Padova, Via Marzolo 8, Padova, 35131 IT Italy; 2 Dipartimento di Biomedicina Comparata e Alimentazione, Università di Padova, Viale dell’ Università 16, Legnaro, 35020 IT Italy

## Abstract

Proteins have coevolved with cellular environments to improve or preserve their functions, maintaining at the same time the degree of hydrophobicity necessary to fold correctly and enough solubility to perform their biological roles. Here, we study the *Escherichia coli* proteome using a Pareto front analysis in the solubility-hydrophobicity space. The results indicate the existence of a Pareto optimal front, a triangle whose vertices correspond to archetypal proteins specialized in distinct tasks, such as regulatory processes, membrane transport, outer-membrane pore formation, catalysis, and binding. The vertices are further enriched with proteins that occupy different subcellular compartments, namely, cytoplasmic, inner membrane, outer membrane, and outer membrane bounded periplasmic space. The combination of various enriching features offers an interpretation of how bacteria use the physico-chemical properties of proteins, both to drive them into their final destination in the cell and to have their tasks accomplished.

## Introduction

All biological systems must efficiently carry out multiple tasks to strive for survival. In some instances, the performance levels cannot be concurrently optimized for all tasks, so that the competition between them affects phenotype selection. Consequently, organisms evolve and adapt themselves to the environment through a precise trade-off. To fully disclose the properties of this complex multi-objective optimization problem, scientists have employed the Pareto front analysis^1–5^. This approach assumes that the process of natural selection promotes phenotypes that trade off their performances among the competing tasks in an optimal way. Each phenotype can be mapped into the space of its physical traits, also referred to as morphospace.

Several observations on living systems show that many phenotypes cluster in small, convex regions of this space^6^. This finding can be interpreted as a signature of a Pareto optimization process, where selected solutions must fall inside convex regions, defined as Pareto fronts. Phenotypes outside the fronts are suboptimal for performing different competing tasks and are thus wiped out by the evolutionary pressure. Phenotypes located at the vertices are called archetypes^7–9^ and are associated with the competing tasks. The performance of each task is optimal at the corresponding vertex and decreases with the distance from it.

Pareto optimization has previously been applied to several biological problems, including human breast cancer^8^, animal behavior and shapes^7^, microbial metabolism^10^, longevity-mass relationship^11^, gene expression^12^, ammonite shapes^13^, and complex networks^14^. Moreover, optimization principles have already been invoked to explain the origin of protein folds^15,16^.

In this paper, we extend the Pareto front analysis to the molecular level. We find evidence that *Escherichia coli* (*E.coli* for short) proteins were selected by trading off the performances of different competing tasks, and we infer the latter ones. According to the Pareto interpretation, we suggest that *E.coli* seems to exploit solubility and hydrophobicity signals to drive the proteins in the cell compartments where they perform the required biological functions at their best. Finally, in the specific case of membrane proteins, which inherently have very low solubilities, our analysis can split apart outer and inner membrane proteins, using their different hydrophobicities.

## Results

### Dataset

We chose *E.coli* as a simple prototype organism since it has been widely studied and, furthermore, its genome is extensively annotated. With the aim of finding coarse-grained attributes of proteins to be used as traits in a Pareto front analysis, we extracted from the Taguchi’s database^17^ the following three continuous characteristics: experimental solubility, experimental yield, and predicted isoelectric point (pI). All quantities were available only for a subset of 3,172 proteins. We added, as a further fundamental continuous trait, an overall measure of protein hydrophobicity, which was obtained by summing up the hydrophobicity values of all its residues according to the Kyte-Doolittle scale^18^.

Three of the above traits inherently convey competing chemical characteristics of polypeptide chains concerning both a water-like solvent and different cellular environments, such as the crowded cytoplasm and the interior of biological membranes. The yield, which is how many proteins are expressed by the ‘*in vitro*’ reconstituted translation system^17^, adds a further characterization.

### Task and environments

We started by extending the state-of-the-art Pareto analysis^7^, in order to connect specific sub-cellular environments with the competing tasks performed by the proteins located in these regions. We made the following assumptions:

(i) The bacterium environments are characterized by specific concentrations, $({\rho }_{1},{\rho }_{2},\ldots ,{\rho }_{n})\equiv \rho$, of *n* chemicals (water, lipids, etc.). As one moves from one place to another, *ρ* varies with continuity at the mesoscopic scale. This is a formal representation of the fact that, even though bacterial cells lack membrane-bounded organelles, they are intricately organized, with different chemical concentrations in different locations^19–21^.
(ii) Each protein can perform *k* possible tasks/activities, and to each of them (the *j*-th task) we may associate a specific performance P_*j*_, as measured by the amount of biological activity of *j*-th type, $j=\mathrm{1,}\ldots ,k$. The *j*-th task is performed at its best in the environment characterized by ${\rho }^{(j)}$, i.e. *P*_*j*_ is maximal at a specific value of *ρ* (e.g. transport is better carried out where there is a high concentration of chemicals that need to be transported from one membrane side to the other). The environment with $\rho ={\rho }^{(j)}$ will be called the *j*-th environment. As a consequence, the performances are in trade-off, since the *k* environments where each of them can be maxized are mutually exclusive (one could also assume that the environments are $k^{\prime}  < k$, since more than one performance can be maximal in the same environment).
(iii) The relevant traits are represented by a vector *v* that targets the protein to the environment characterized by *ρ*(*v*), in such a way that its *biological function* is maximally exploited. Thus the *j*-th performance is assumed to be a function of *p*(*v*), ${P}_{j}(\rho (\nu ))$.
(iv) The biological function of a protein is quantified by its *fitness* function, as follows:

1 $$F({P}_{1}(\rho (\nu )),\ldots ,{P}_{k}(\rho (\nu )))\mathrm{.}$$

*F* is assumed to be an increasing function of all its arguments. According to (*iii*), we must maximize *F* with respect to *v* in order to find where the protein characterized by *F* will be directed. The derivative of *F* with respect to the traits *v* leads to the optimal solutions:

2 $$0=\frac{\partial F}{\partial {\nu }_{m}}=\sum _{j\mathrm{=1}}^{k}\frac{\partial F}{\partial {P}_{j}}\frac{\partial {P}_{j}(\rho )}{\partial {\nu }_{m}}\mathrm{.}$$

From (*ii*) *P*_*j*_(*ρ*) is maximum at $\rho ={\rho }^{(j)}$. We make the simplifying hypothesis that ${\rho }^{(j)}\equiv \rho ({\nu }^{(j)})$ and, at the leading order in $\rho -{\rho }^{(j)}$,

3 $${P}_{j}(\rho )={P}_{j}({\rho }^{(j)})-{(\rho -{\rho }^{(j)})}^{T}g(\rho -{\rho }^{(j)}),$$

Where *g* is some metric tensor and, at the leading order in $\nu -{\nu }^{(j)}$,

4 $$\rho (\nu )-\rho ({\nu }^{(j)})=M(\nu -{\nu }^{(j)}),$$

With ${M}_{i,m}=(\partial {\rho }_{i}(\nu )/\partial {\nu }_{m}{)}_{\nu ={\nu }^{(j)}}$. This leads to

5 $$\mathrm{0=}\sum _{j\mathrm{=1}}^{k}\frac{\partial F}{\partial {P}_{j}}\hat{g}(\nu -{\nu }^{(j)}),$$

where $\hat{g}={M}^{T}gM$ is the induced metric tensor in trait space. Thus, we are led to the condition for the optimal choice of *v*,

6 $$\nu =\frac{\sum _{j\mathrm{=1}}^{k}{\nu }^{(j)}\partial F/\partial {P}_{j}}{\sum _{j\mathrm{=1}}^{k}\partial F/\partial {P}_{j}}\,,$$

which means that the optimal *v* lies in the convex hull in *v*-space whose vertex are *v*^(*j*)^, $j=\mathrm{1,}\ldots ,k$. We then expect that a convex hull in the trait subspace is a signature of a Pareto optimization in the *E.coli* proteome.

### Morphospace analysis

With each protein represented by the set of continuous traits defined above, and with the above derivation in mind, we apply a Principal Component Analysis (PCA) to reduce trait vector dimensionality and search for Pareto polytopes. The PCA variance is mainly explained (about 95%) by two principal components that are substantially parallel to the hydrophobicity (PC1) and solubility (PC2) trait, respectively (Table 1, Fig. S2). This can be rationalized by considering that hydrophobicity is the dominant force implicated in the folding process of globular proteins^22–25^, whereas solubility is a property that emerges as a necessary feature to prevent protein aggregation^26–28^, and, consequently, the onset of relevant maladies in humans^29^. Solubility also appears to be related to mRNA expression levels, at least for specific proteins^30^. The maintenance of protein solubility is also a fundamental aspect of protein homeostasis^28^, being an essential requirement for protein functionality. Furthermore, proteins are evolutionarily selected to perform necessary and useful functions, so they must be stable (at least marginally) but also flexible enough to accomplish their tasks through relevant conformational changes.

**Table 1:** Principal components and their relative weights.

Table Of Loadings	PC1	PC2	PC3
Hydrophobicity	**0.9996**	0.0002	0.0275
Solubility	−0.0040	**0.9999**	0.1409
Yield	−0.027193	−0.1410	**0.9896**
Calculated pI	0.0037	−0.0069	−0.0095

**Figure 1:**
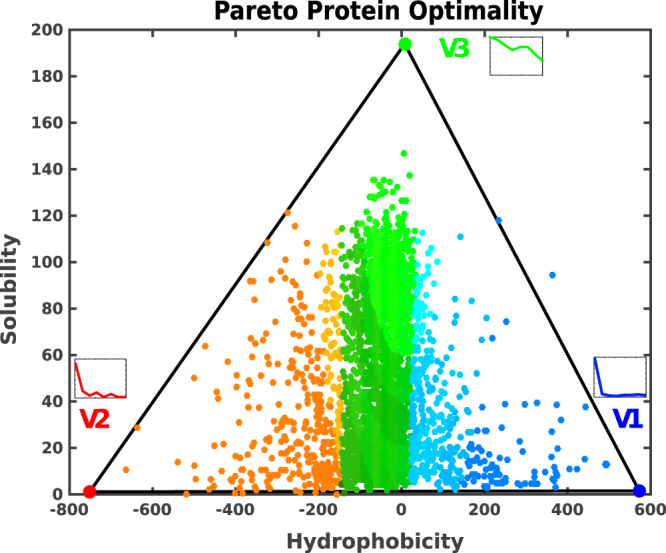
Solubility-hydrophobicity triangle. We show a scatter plot of the 3,172 proteins of the *Escherichia coli* proteome. Each protein is represented as a point whose coordinates are the values of its hydrophobicity and solubility. The Pareto front is the triangular-hull that exhibits a low p-value of the order of 5 · 10^−3^, confirming the statistical significance of the plotted distribution (see the Supplementary Information for more details). Proteins whose points lie inside the triangle are the best compromise in the multi-objective optimization of the three tasks, which are better performed by the corresponding archetypes located at the three vertices. Points outside the triangle would have a better counterpart inside the triangle in at least one of the tasks. The RGB colors identify the distribution of the integral inner membrane (blue), outer membrane, and outer membrane bounded periplasmic (red) and cytoplasmic (green) proteins, which also characterize the vertices.

In the solubility-hydrophobicity space, the *E.coli* proteins lie inside a triangle, a clear hallmark of Pareto optimality (Fig. 1). The statistical significance of the Pareto front is assessed using the t-ratio test^7^, which evaluates the ratio between the area of the convex hull and the area of the minimum triangle in which the convex hull can be embedded. The t-ratio of the experimental data points is then compared to the t-ratios of 10^4^ null-models (generated by the original data distributions). The p-value, which is a function of the t-ratios, is lower than 5*10^−3^ (see Methods). If we z-score solubility-hydrophobicity-yield-pI traits before PCA, we find that the variance changes with the pI trait, which this time becomes relevant. However, by projecting the data points in the first two principal components, as obtained from the z-scored traits, the resulting convex hull is not a triangle anymore, with a p-value >0.05, as evaluated from the t-ratio test.

### Enrichment analysis: subcellular localization

The enrichment of specialized tasks of the vertices defining the convex hull that encloses all the data points is the second signature of a Pareto optimal front. When a vertex enrichment analysis is carried out, considering the subcellular localization labels, as obtained from the Taguchi’s dataset^17^, a strong signal emerges. The vertices with the lowest solubility values are mainly populated by membrane proteins (V1 and V2 in Table 2). Nonetheless, there is a clear-cut distinction between the two vertices. Vertex V1 has a very high hydrophobicity component, in the trait vector, and is enriched in inner membrane proteins (represented by blue points in Fig. 1). Whereas vertex V2, which presents higher water-like propensity (i.e., low hydrophobicity), is enriched in outer-membrane and outer membrane bounded periplasmic proteins (red points in Fig. 1). This sharp separation between membrane proteins (both with low solubilities) is striking, and it shows that the different values in their hydrophobicity component appear to be an essential ingredient in driving membrane proteins to their final destination. Vertex V3, which has a very high solubility, is enriched with proteins that occupy the cytoplasmic region (green points in Fig. 1).

**Table 2:** Inferred tasks for each archetype in the *Escherichia coli* proteome, along with subcellular localization labels.

Archetype (Vertex)	Inferred tasks	Subcellular localization	Enriched GO-annotations
			Cation transmembrane transporter;
Blue (V1)	Transporting	Integral Membrane	Active transmembrane transporter;
			Anion transmembrane transport.
Red (V2)	Polysaccharyde, Binding, Catalysis	Outer Membrane and Outer Membrane Bounded Periplasmic	Porin activity;
			Polysaccharide metabolic process;
			Hydrolase activity;
			Molecular function regulator.
Green (V3)	Regulation	Cytoplasm	Regulation of the metabolic process;
			Regulation of biological process.

### Enrichment analysis: GO annotations

The distribution of Gene Ontology annotations^31^, considered as a function of the distance from the polytope vertices (the archetypes), unveils the competing tasks related to them. The Gene Ontology annotations of each protein hereafter referred to as GO-terms, are extended to include the parent GO-terms, to improve the robustness of protein annotations (see SI for further details). We bin the space into equally populated regions^8,11^, and for any given annotation, we check whether the first bin is more enriched than the other bins. The statistical significance of the enriched terms is evaluated with a Benjamini-Hochberg procedure to take into account the problem of multiple hypothesis testing. Finally, the False Discovery Rate (FDR) with a threshold set to 0.05 is computed (see SI).

**Figure 2:**
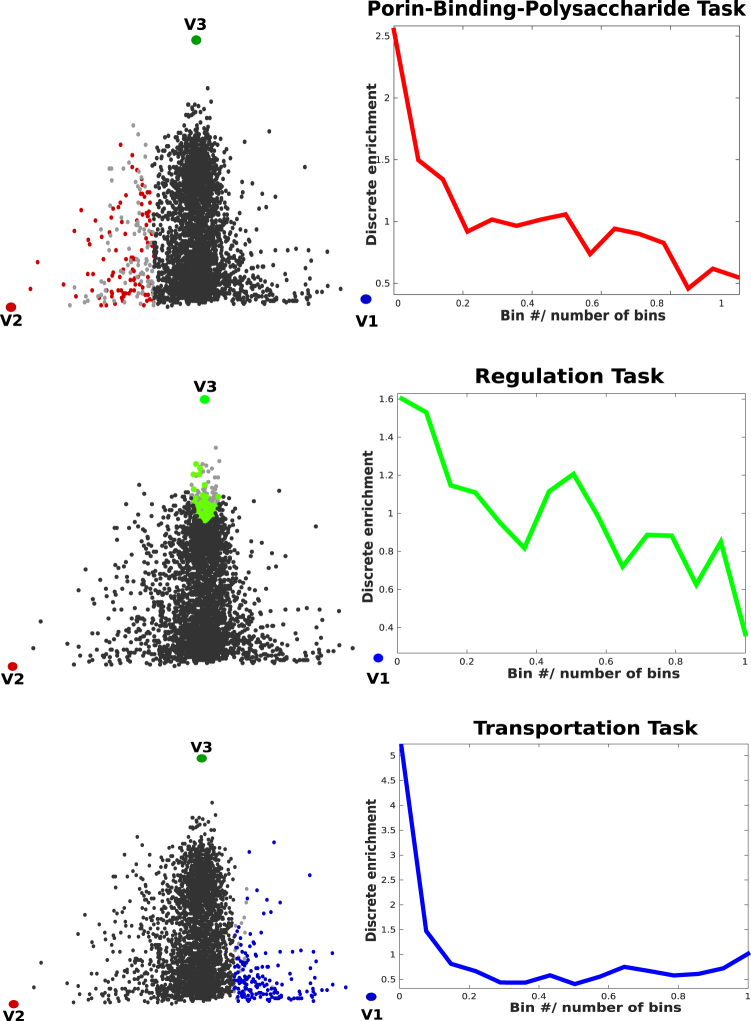
Enrichments. Enrichment plots as a function of the distance from the corresponding archetype. Pareto optimality is defined such that the points closest to the vertices of the triangle must be maximally enriched in some features (they behave as specialists or “pure” types). All the tasks (GO-terms) that enrich each vertex are added together. Next to the enrichment plot, the proteins are mapped in the solubility-hydrophobicity plane. The colors highlight the enriched proteins belonging to the first bin. The vertices in the figures (V1, V2 and V3) label the protein subcellular localizations (as presented in Fig. 1), namely, cytoplasmic proteins (green), integral inner membrane proteins (blue), outer membrane, and uter membrane bounded periplasmic proteins (red).

Based on this analysis, we find GO-annotations that are significantly enriched at each vertex. The vertex V1 (blue) is enriched in transmembrane transporters; in the vertex V2 (red) we observe enriched GO-terms for Porin activity, polysaccharide metabolic process, and hydrolase activity; the third vertex V3 (green) is enriched in molecular functions related to different kinds of regulation tasks. The enrichment densities of these features are shown in Fig. 2 and listed in Table 2.

According to our mathematical derivation, the tasks found to enrich the triangle vertices are expected to be better performed in the distinct subcellular localizations that label the corresponding vertices. This finding is confirmed by the types of GO-terms, related to the molecular functions and biological processes, that enrich those vertices.

### Evidence for a tetrahedron

**Figure 3:**
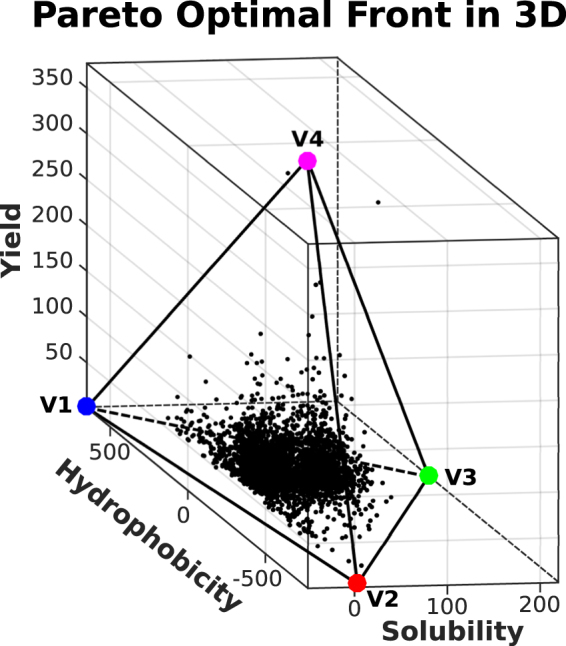
Tetrahedron in the hydrophobicity-solubility-yield space. The three vertices in the hydrophobicity-solubility plane correspond to the archetypes identified in the previous subsection.

When the Pareto analysis is extended to include protein yield, a tetrahedron emerges as the convex hull representing the new front in *3D* (Fig. 3). The yield feature, as derived from the Taguchi’s dataset, corresponds to the third principal component (see Table 1). The tetrahedron encloses most of the data points, with a p-value smaller than 0.01%. Based on the Pareto theory, all the vertices of the tetrahedron must be enriched with at least one feature per vertex, in order to infer the competing tasks for all the vertices. The triangular convex hull discussed above can be obtained from the tetrahedron by projecting it on the solubility-hydrophobicity plane, so that the enriched features found for triangle vertices can be associated to three of the tetrahedron vertices as well.

The new vertex, V4, is characterized by proteins with a high yield component, low hydrophobicity, and low solubility. This vertex, similar to vertex V3, is enriched with cytoplasmic proteins; however, the tasks that characterize vertex V4 are different. According to our GO-terms analysis (see Fig. S19), they are related to RNA processes such as tRNA metabolic process (GO:0006399), tRNA modification (GO:0006400 and GO:0009451) and ncRNA metabolic process (GO:0034660). This finding indicates that proteins involved in tRNA/RNA metabolic processes are also the ones that have higher expression levels in a cell-free translation system. However, in contrast to the two-dimensional triangular Pareto front, the found tetrahedron is not robust. When few data points with the highest yields are removed (see SI), the p-value increases from 10^−4^ to 10^−1^, making the results of this analysis less reliable.

## Discussion

From a general perspective, our results broaden the scope of the Pareto analysis with respect to the state-of-the-art approaches^7^. Pareto polytopes have been shown to enclose the variation of phenotypic traits for organisms of the same species that adapt to different environmental niches, or the variation of gene expression patterns for cells of the same organism that adapt to different tissues (or pathological conditions in the case of tumor cells). In this paper, we extend the Pareto front analysis to a further downward step toward shorter scales, by showing that the variation in protein physico-chemical features can be explained as the result of a multi-objective adaptation to different sub-cellular compartments, to optimize the related biological tasks. Concurrently, we find evidence that *E.coli* proteins were selected by trading off the performances of various competing tasks.

According to the standard view, the basic physical properties considered here, hydrophobicity and solubility, were evolved in the first place to allow the foldability of proteins and to prevent them from aggregation. On top of that, our findings suggest the novel idea that the solubility-hydrophobicity signal encoded in the protein sequence can flag the final localization of the latter in the cell, and at the same time can hint at its biological function. According to the Pareto interpretation, the two traits have evolved to optimize three different performances simultaneously, each related to a separate cellular compartment.

**Figure 4:**
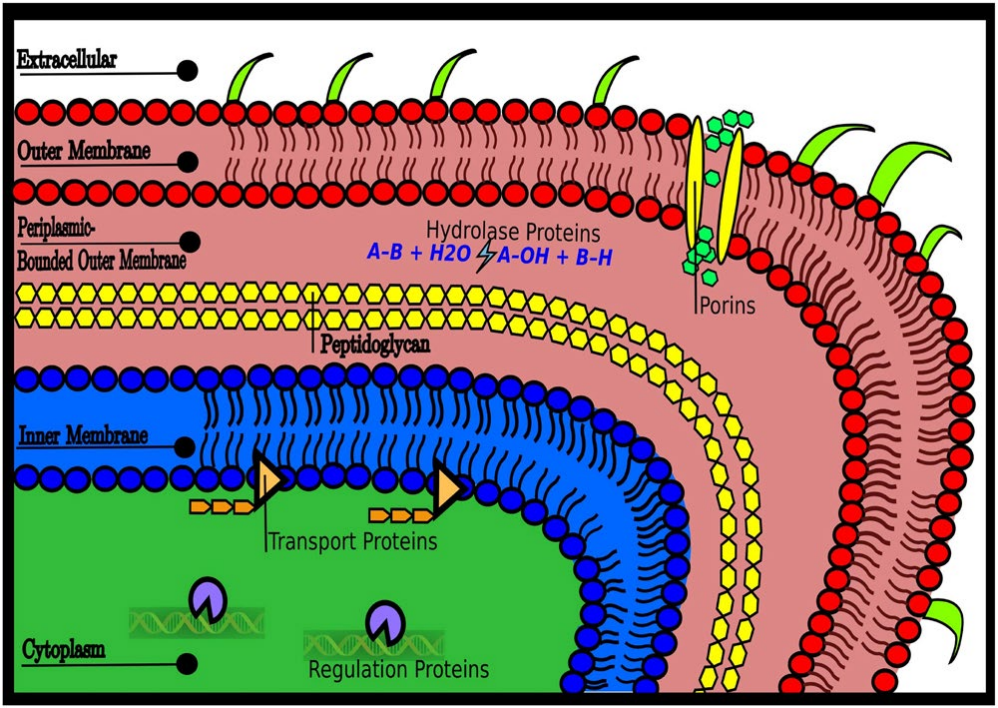
Cell compartments and Pareto triangle. There is a direct mapping between the four different compartments of Escherichia coli (outer membrane and outer membrane bounded periplasmic proteins, inner membrane, and cytoplasm) and the proteins that populate the vertices of the Pareto front.

Thus, the major result of our study is the crucial role played by subcellular compartments in the fitness of the *Escherichia coli* proteome, obtained by a direct mapping between the Pareto front vertices and the subcellular compartments (Figs 2 and 4). It turns out that natural selection pushed the bacterium to optimality by tuning the solubility-hydrophobicity traits of all proteins, in such a way that each of them can reach the distinct environment where it can perform the required task at its best. On the other hand, protein biological tasks are eventually related to their interactions with metal ions, ligands, substrates, other proteins, or nucleic acids. Therefore, one could speculate that the specific solubility-hydrophobicity traits of each protein are needed to optimize the interactions associated with the related biological tasks.

The Pareto analysis shows that the protein performances are in a trade-off with each other and identifies archetypal tasks located closer to polytope vertices. From that, we can infer that the archetypal proteins found at vertex V1 of Fig. 1 (inner membrane) are specialized in the transport of organic and inorganic molecules. Archetypal proteins at vertex V2 (outer membrane and periplasmic space) are specialized in wide-pore forming from the intake of molecules, catalysis, binding activity and polysaccharide metabolic processes, while those at vertex V3 (cytoplasmic space) are specialized in the regulation of different processes (Table 2). As noted before, the difference in solubility can be due to different structural classes^17^. Nonetheless, we found that membrane proteins, which have very low solubility (also confirmed by experimental data^17^), can be split into outer and inner membranes through their hydrophobicity. Notably, the two membrane protein classes have very different structures, even though their measured solubility is similar.

If protein yield is added as a third trait to the Pareto front analysis, a statistically significant tetrahedron emerges as the convex hull enclosing all data. The tetrahedron base, in the hydrophobicity-solubility plane at the low yield, reproduces the already discussed triangle with vertices V1, V2 and V3 corresponding to different cellular compartments. The fourth tetrahedron vertex, V4, at high yield, is inferred to be related to archetypal proteins that are cytoplasmatic (as for vertex V3) but involved explicitly in tRNA/RNA metabolic processes. The finding that proteins highly expressed by a cell-free translation system^17^, based on translation factors, tRNAs and ribosomes, with no chaperons involved, can be associated to Pareto optimality through their functional role in tRNA/RNA metabolic processes is intriguing. In keeping with the general framework established in this work, whereby different tasks are associated with different environments, the presence of RNA molecules may be interpreted as defining a specific type of environment for the archetypal V4 protein.

The problem of spatial protein distribution in bacteria is of paramount importance since the subcellular localization of proteins is crucial to provide the physiological context for their function, to achieve functional diversity and to economize protein design and synthesis^32^. Although bacterial cells (such as *E. coli*) lack internal membrane-bounded structures, they are not “bags of mostly randomly localized macromolecules”^19^. Instead, they are organized with different macromolecules that display complex subcellular localization patterns^20,21,32^. Different mechanisms drive proteins toward their final cell destination^20,21,32^ through the cytoplasm and the subcellular localization of proteins in *E. coli* across the different membrane barriers, and one of the major achievements that our analysis offers is a significant breakthrough for the comprehension of this transport mechanism. With the Pareto front analysis, we find indications that Gram-negative bacteria exploit the solubility and the hydrophobicity of proteins to take them in the major compartments where they can perform the function needed for the organism at their best. This finding does not exhaust the complexity of the protein sorting, but it adds new clues. Among all known mechanisms and signals, the solubility-hydrophobicity balance of a protein could be exploited by the cell as a subcellular localization signal. According to our results, it appears that solubility and hydrophobicity values provide a signature to the protein’s final destiny, and possibly an indication of the task that proteins perform at their best in that environment. This result, which was obtained from our Pareto analysis, should be experimentally validated in future research.

## Methods

### Principal Convex Hull Analysis (PCHA)

We performed the archetypal analysis, introduced by Cutler and Breiman^4^, whose goal is to find the best-fitting convex hull of the data in the trait space, that is the solution of the minimization problem (see eq.^7^). This can be done computationally by the PCHA algorithm, developed by Morup *et al*.^5^ and implemented in the Pareto Task Inference (ParTI) developed by Hart *et al*.^8^. This algorithm allowed us to find the explained variance of the convex hull that encloses the data points, as a function of the number of vertices (see Fig. S3). The positions of the vertices of the convex hull in the trait space were determined by employing the Sisal algorithm^33^ which is analogous to PCHA but considers in a more flexible way the presence of outliers and the possibility that archetypes lie outside the convex hull^8^. See Table S2 for the archetype positions found using Sisal, after 100 iterations, and Fig. S5 for the archetype positions using different types of algorithms. We also computed the errors in the positions of the archetypes by employing the so called bootstrapping method^8^. This relies on the generation of n-bootstrapped datasets with the same number of proteins (3,172) as the original dataset, and on computing from each new dataset the corresponding archetype positions. We generated 10^4^ bootstrapped datasets, and we computed their center of mass and the standard deviations of archetype positions. Errors are depicted as ellipsoids in Fig. S4.

### Statistical significance and robustness of the Pareto fronts

We computed the p-value to measure the statistical significance of the detected Pareto front polytope. The p-value computation is based on the t-ratio, which is defined as the ratio between the volume of the polytope, which is the triangular convex hull with three vertices found in Fig. 1, and the volume of the convex hull with a higher number of vertices that encloses the majority of the data points. The t-ratio is usually larger than 1, and the closer it is to 1, the better the polytope captures the shape of the data. After computing the t-ratio on the original dataset, we compared it with the t-ratio derived from *n* null models, obtained by randomizing pairs of solubility and hydrophobicity values from the original data, i.e., by taking the same cumulative distribution function (CDF), along single axes, as in the original dataset. The p-value is then defined as the fraction of null-models with a t-ratio lower than the original one. The high statistical significance is generally associated to p-values lower than 5%. Pareto analysis can be hampered when the results are heavily influenced by the presence of some outliers (see Fig. S6). Statistically speaking, the results must be, as much as possible, outlier-independent. More practically, the deletion of a small number of data points in the above analysis must not affect archetype identification and the p-value of the detected polytope. We generated 10^4^ null-models for all of the six possible combinations of the four continuous traits, finding that the most robust triangles with the lowest p-values are projected in the hydrophobicity-solubility and hydrophobicity-yield planes (p-value of the order of 0.5%). In the remaining four cases the lowest p-value is higher than 5%. We further found that the triangle in the yield-hydrophobicity plane is strongly dependent on outliers, while the triangle in the solubility-hydrophobicity plane is very robust. In the former case, the p-value fluctuates in the range 0.5–10% when (up to 4) proteins with the highest yield are removed, while in the latter case the p-value is almost unaffected (see Fig. S6).

## Electronic supplementary material

Supplementary Materials

Main Dataset
