# Supplementary material for: Signature of Pareto optimization in the Escherichia coli proteome
Source: Sci Rep. 2018 Jun 14;8:9141. doi: 10.1038/s41598-018-27287-3 (PMC6002381; doi:10.1038/s41598-018-27287-3)
Supplement: Supplementary file 1 — Supplementary Materials [file 41598_2018_27287_MOESM1_ESM.pdf]

Supplementary Materials for

Signature of Pareto optimization in the *Escherichia coli* proteome

L.Koçillari[1], P.Fariselli[2], A.Trovato[1], F.Seno[1], A.Maritan[1]

1. INFN and Dipartimento di Fisica e Astronomia G. Galilei, Università di Padova, Via Marzolo 8, 35131 Padova, IT.

2. Dipartimento di Biomedicina Comparata e Alimentazione, Università di Padova, Viale dell'Università 16, 35020 Legnaro, IT.

Correspondence to: amos.maritan@pd.infn.it

This PDF file contains Methods and Materials on:

Archetype density analysis (Results) 8
Evidence for a Tetrahedron 19
References 21

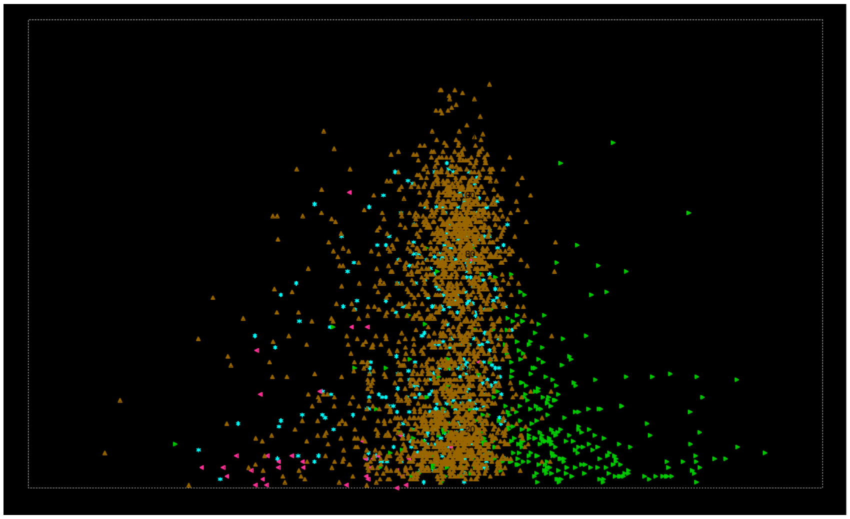

Figure 1: The Pareto front. Data points in the space of solubility vs hydrophobicity. Proteins are coloured as follows. Green:Inner membrane, Yellow:Cytoplasmic, Light blue:Periplasmic-bounded outer membrane, Rose:Outer membrane.

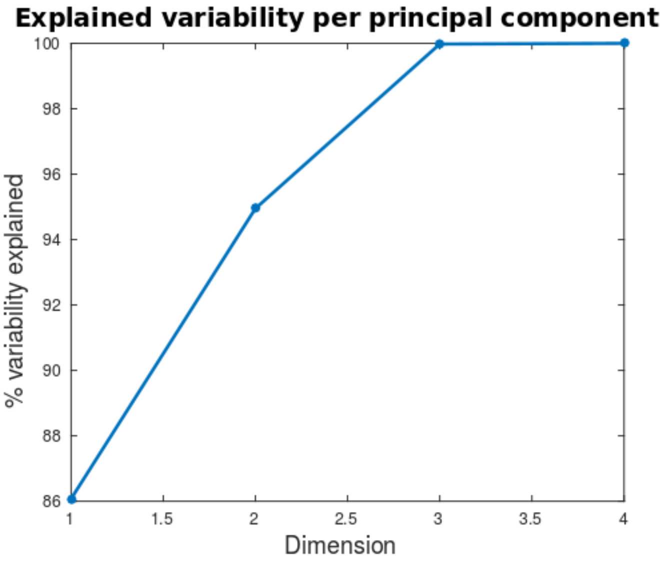

Figure 2: PCA for the four dimensional space of continuous traits. The first component is better explained by the hydrophobicity, the second component by the solubility, whereas the third component by the protein yield (see Table 1). The first two traits, i.e. solubility and hydrophobicity, are able to explain around 95% of the overall variability. We achieve almost the total variability if we consider also the third principal component, but in this three dimensional morphospace the convex hull is affected by robustness caveats (see Section 3.2).

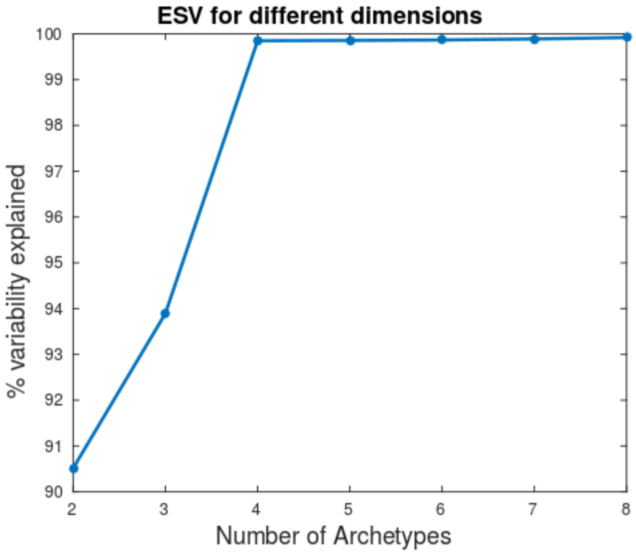

Figure 3: Number of archetypes. Explained variance (*1*) of the data points as a function of the number of archetypes. In our analysis, we considered only the first three archetypes, which account for 94% of the total variance.

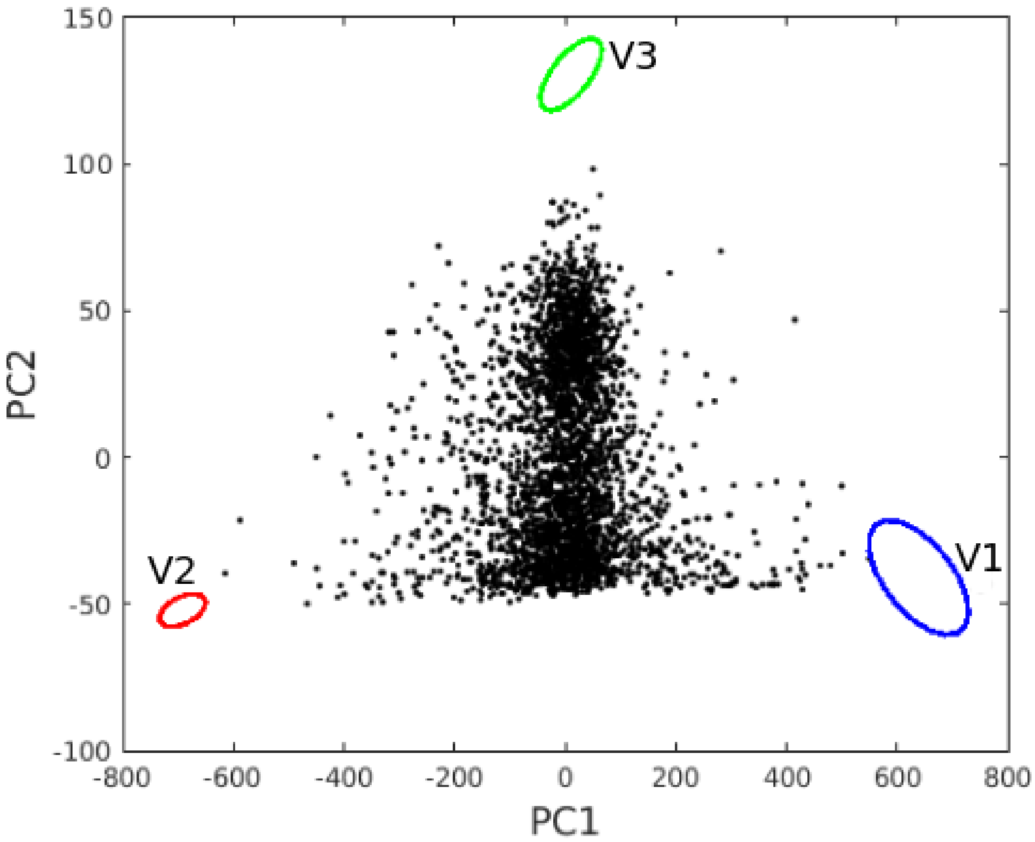

Figure 4: Archetype positions. Error distribution of the coordinates of the vertices of the triangle as obtained by the Sisal algorithm (2) performing 10^4 bootstrapped datasets .

Arch (PCA) Position	Hydrophobicity (PC1)	Solubility (PC2)
Blue	639.2	-41.0
Red	-691.6	-52.2
Green	10.9	130.5

Arch (Orig) Position	Hydrophobicity	Solubility
Blue	572.4	1.5
Red	-751.7	1.1
Green	7.3	193.9

Table 1: Position of the three archetypes as found with Sisal. The positions of the three vertices in the principal component plane are shown in the top table, whereas the same positions in the solubility -hydrophobicity plane are shown in the bottom table.

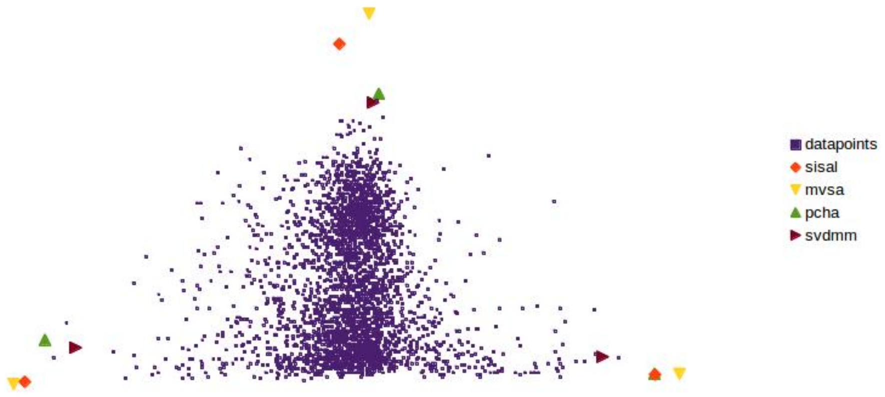

Figure 5: Archetype coordinates. Archetype coordinates evaluated with four different methods such as Sisal, PCHA, MVSA, SVDMM. They give equivalent results.

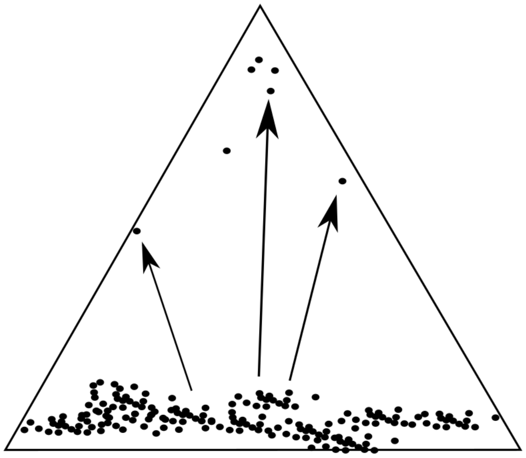

Figure 6: Robustness of the Pareto front. PCHA analysis does not necessarily imply that the data are well distributed on a convex hull. Sometimes Pareto analysis cannot be applied, for example in cases where the outliers dominate the statistics and triangles appear even when the majority of points clusters only in specific regions of the convex hull and a few outliers are responsible for adding other vertices.

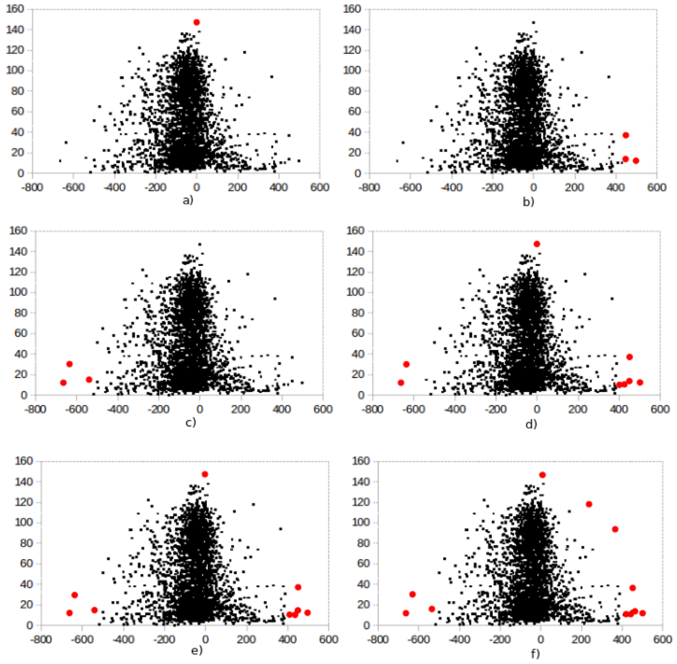

Figure 7: Robustness of the triangle in the solubility vs hydrophobicity plane. We computed the p-value, after removing the proteins in red, for each case. For a) p-value= 0.5%, b) p-value=0.4% , c) p-value$<$ 0.01%, d) p-value=0.06%, e) p-value= 0,04%, f) p-value$<$ 0.01%

1 Archetype density analysis (Results)

Pareto optimization analysis is based on the following conditions:

- The first condition demands for the clustering of the majority of data points within a well-shaped polygon or polyhedron (in our case we find that data points cluster into a well-shaped triangular-hull with a p-value of the order of $5 * 10^{-3}$).
- The second condition is related to the density analysis of the archetypes/vertices. Each vertex must be enriched with at least one discrete or continuous feature characterizing the corresponding archetype. Density profiles of the features enriching a given vertex must attain their maximum value in the region (or bin) of the polytope containing that vertex, and then decrease monotonically with the distance from it.

Based on these conditions, Pareto optimality theory allows us to infer competing tasks for each vertex of the polytope (three tasks in our triangular case) from the attributes of the corresponding enriched features (continuous or discrete).

1.1 Enrichment analysis with continuous and discrete features

Enrichment analysis was performed on additional discrete features assigned to each data point, such as the subcellular localization annotations (6 annotations), obtained from the Taguchi's dataset, and the GO-annotations (702 annotations). GO-annotations were obtained from the Gene Ontology dataset (*3*) which has the structure of a directed acyclic graph with nodes, called GO terms, which describe the molecular functions of each protein, their locations in the cell environment and the biological processes in which they are involved. Below, we will show how to build the complete table of discrete features for the enrichment analysis.

We treated the discrete features on the same footing as the continuous features, by assigning to data points the value 1 if they hold a given feature and 0 otherwise. For each vertex we associate a ranked vector of euclidean distances ordered from the nearest point to the furthest from the vertex. Data points are then clustered in bins, such that each bin has the same number of points. We compute the ratio of densities of the discrete feature in a given bin, with respect to the mean density among all data. The results, plotted versus the bin number (ordered from the nearest to the farthest from the archetype), are shown in Figure 8 (see also Figure 2 in the main text).

1.2 Statistical significance of enriched features

The statistical significance of the enriched features can be evaluated by computing a p-value test, based on the probability of finding a higher density of the feature in the first bin with respect to the other bins (see supplementary materials (*4*)).

We analyzed a large dataset of 708 discrete features. With such a big number, several enriched curves could appear just by chance. Thus, the p-values must be corrected for the

possibility of false-positive p-values. A common approach employed to deal with these type of errors is the false discovery rate (FDR) (*5*).

The statistical significance of enriched features was tested also against the null-model, by reshuffling the values of a given feature. It is expected that only a few enrichments survive after a random reshuffling. For 10^3 random datasets, with 708 randomized features each, we found that only 50 out of 10^6 NULL-features are enriched by chance, with a threshold of 0,05 for the FDR.

1.3 Sub-cellular Localization Annotations

The process of targeting proteins towards the correct cellular compartments seems critical in the functionallity of prokaryotes and eukaryotes. Here, we are looking for optimization criteria which drive the localization of proteins inside the cells. As pointed out in the above section, Pareto optimization requires enriched features at the archetypes, so that we consider as discrete features the sub-cellular localization annotations as given by Taguchi (*6*). Each protein is labelled with one out of eleven possible cellular component features: periplasmic, cytoplasmic, inner membrane, outer membrane beta barrel (see figures 1 and 3 in the main text), membrane anchored, inner membrane lipoprotein, outer membrane lipoprotein, membrane lipoprotein, membrane associated, perisplasmic with N-terminal Membrane Anchored and extracellular proteins. We selected for further analysis only the six features with an occurrence frequency higher than 15: periplasmic, cytoplasmic, inner membrane, outer membrane (see Figure 3 in the main text), membrane anchored, outer membrane lipoprotein.

We remind that in *Escherichia coli*, as in other gram-negative bacteria, the cytoplasm is surrounded by a multi-layered cell envelope that consists of the plasmatic or inner membrane, composed of a phospholipid bilayer, and a second external lipid bilayer, identified as the outer membrane. This second external membrane is asymmetric and has a different composition with respect to the inner membrane. Moreover, the outer membrane exposes lipopolysaccharide molecules to the external environment. The outer membrane, is the most protective barrier for the organism, and the lipidic layer, together with the outer membrane proteins and the lipopolysaccharide, create the tactile organ of the gram-negative bacteria. Between the two membranes lies the periplasm, a crowded space that contains proteins, small molecules and a peptidoglycan mesh layer (*7*)

From the analysis of the density profiles we find that each archetype/vertex is enriched with at least one sub-cellular location. In particular, the left vertex (with low solubility, low hydrophobicity) is characterized by a high density of outer membrane and periplasmic proteins. At the right vertex (low solubility and high hydrophobicity) there is an abundancy of inner membrane proteins, while at the top vertex (low hydrophobicity and high solubility) are located more cytoplasmic proteins (see Figure 8). Enrichment curves are rather smooth in the case of a small number of bins ($5 - 10$) while their roughness increase with a higher number of bins.

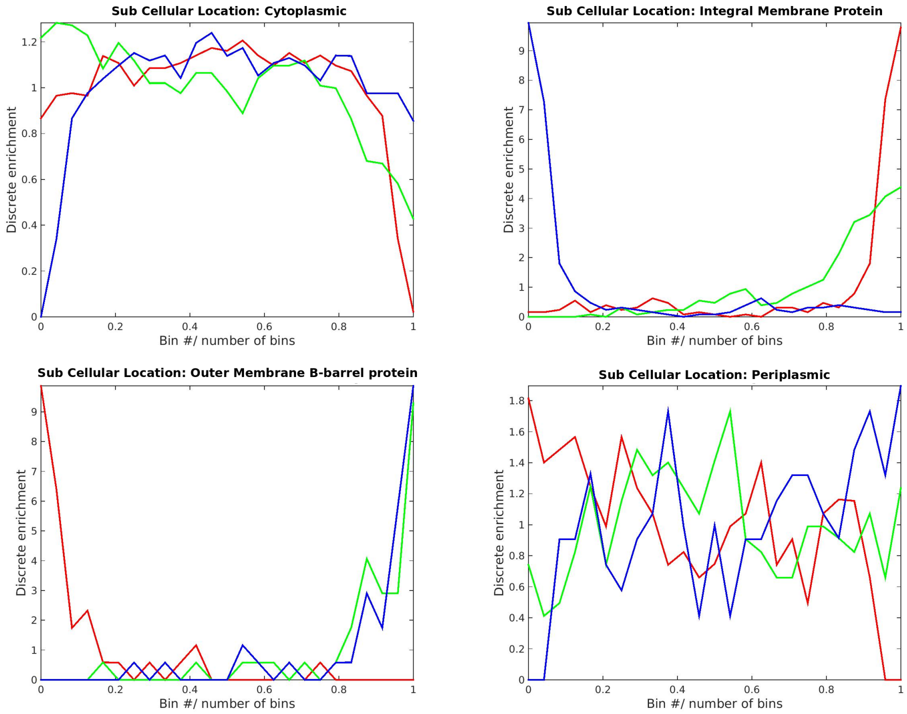

Figure 8: Discrete enrichments of proteins annotated with sub-cellular compartmentalization. Data points are clustered in 25 bins with the same number of proteins according to their euclidean distance from one of the three archetypes. We booleanized the data (1 for proteins with the given feature, 0 otherwise) and for each of the 25 bins we computed the ratio between the fraction of proteins with the specified feature in the bin over the fraction with the same feature inside the whole triangle. This procedure is repeated for all the archetypes. The red and blue curves are almost specular since the triangle is approximately isosceles, with a slight shift toward the blue vertex.

1.4 Gene Ontology Annotations

In this section we show the density enrichment results for the gene-expression of the *Escherichia coli*. We consider the Gene Ontology dataset as given from http://geneontology.org, which consists on a total number of 4442 GO-terms. We booleanized this dataset by assigning to each protein the value 1, if they are annotated with the given term, and 0 otherwise. Then, we considered only those annotations with occurrencies higher than 15, resulting with a final table of 702 GO-terms. (**See Supp. Table**) Each protein can be annotated with more than one GO-term at the same time.

From the analysis of the density profiles, we found that archetypes/vertices are enriched in GO-annotations. In the following section we show the most characteristic GO-terms, from which we are able to unveil the competing tasks associated to each vertex.

1.4.1 Archetype 1

The right vertex, the blue one, is enriched with inner membrane proteins, which are characterized by low solubility and high hydrophobicity. It is highly populated by proteins specialized in the *transportation process* such as: cation transmembrane transporter activity (GO:0008324), ion transport (GO:0006811), active transmembrane transporter activity (GO:0022804), ion transmembrane transport (GO:0034220),ion transmembrane transporter activity (GO:0015075), organic anion transport (GO:0015711), substrate-specific transmembrane transporter activity (GO:0022891). Further GO-terms that specify the inner membrane location are the following: single-organism transport (GO:0044765), intrinsic component of plasma membrane (GO:0031226), single-organism localization (GO:1902578), bacterial inner membrane (GO:0005886) (see Figures 9 and 10).

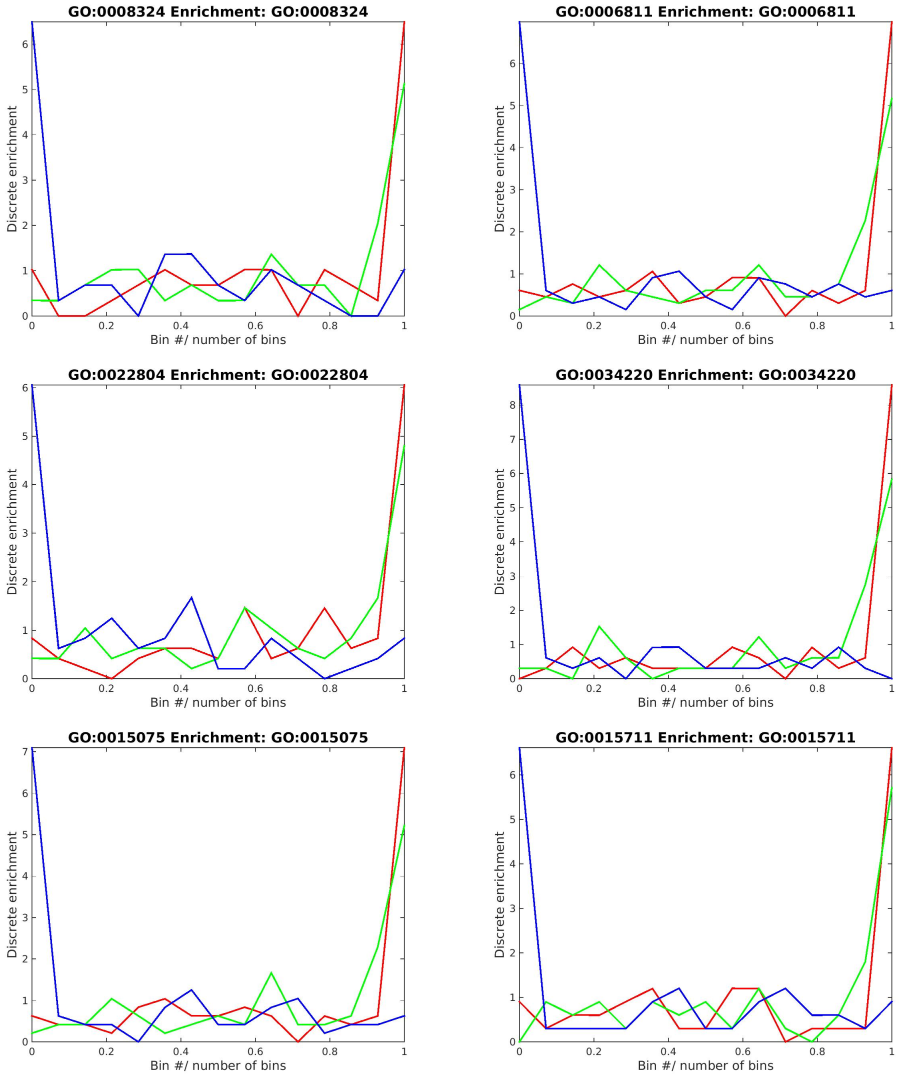

Figure 9: Right Vertex Density enrichments are shown in the case of 15 bins and FDR<0.05.

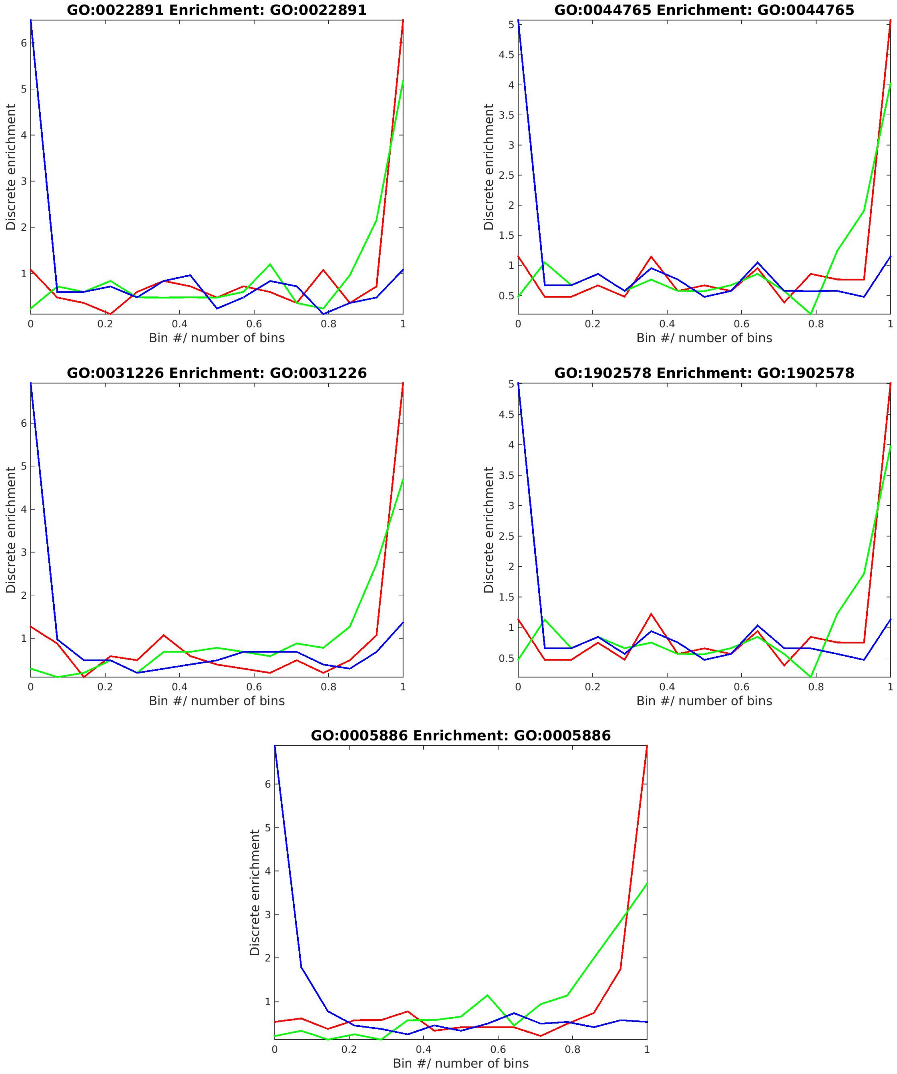

Figure 10: Right Vertex Density enrichments are shown in the case of 15 bins and FDR<0.05.

1.4.2 Archetype 2

At the left vertex, the red one, we find outer-membrane and outer membrane-bounded periplasmic proteins, which are characterized by low solubility and low hydrophobicity. In this vertex, proteins are specialized in *wide-pore forming* from the intake of molecules, *catalysis, binding activity and polysaccharide metabolic processes*. The enriched GO-terms are the following: elemental activities, such as catalysis or binding (GO:0003674), polysaccharide metabolic process (GO:0005976), macromolecule catabolic process (GO:0009057), hydrolase activity (GO:0016787), external membrane of Gram-negative bacteria (GO:0019867), outer membrane-bounded periplasmic space (GO:0030288), cellular polysaccharide metabolic process (GO:0044264), external encapsulating structure part (GO:0044462), 4 iron, 4 sulfur cluster binding (GO:0051539) (see Figures 11 and 12).

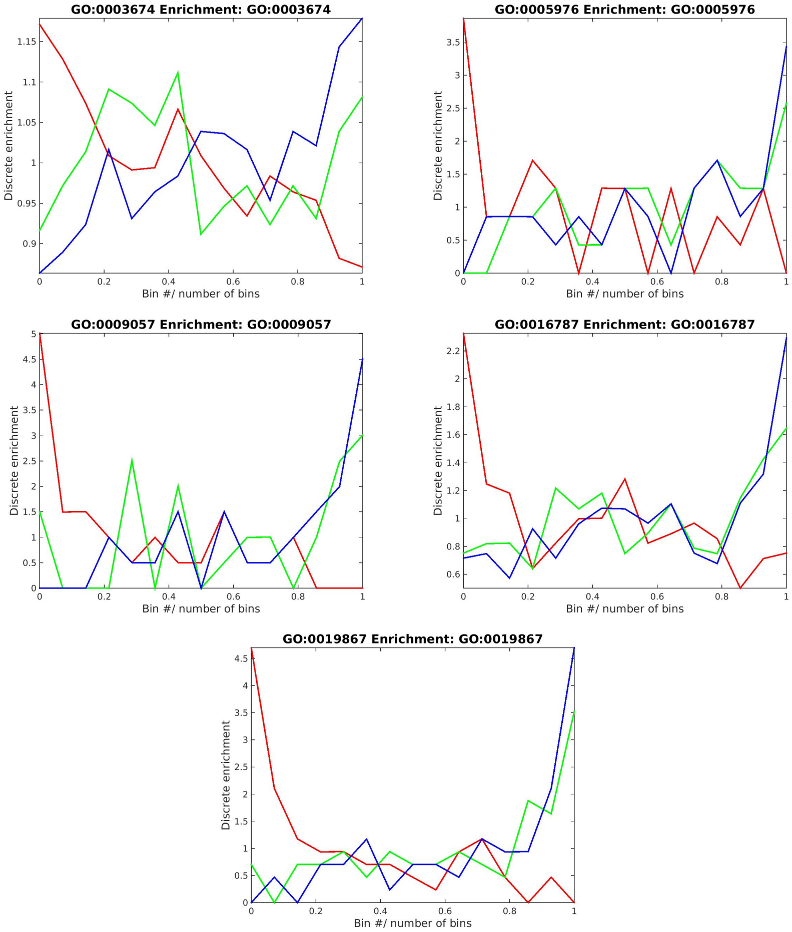

Figure 11: Left Vertex Density enrichments are shown in the case of 15 bins and FDR<0.05.

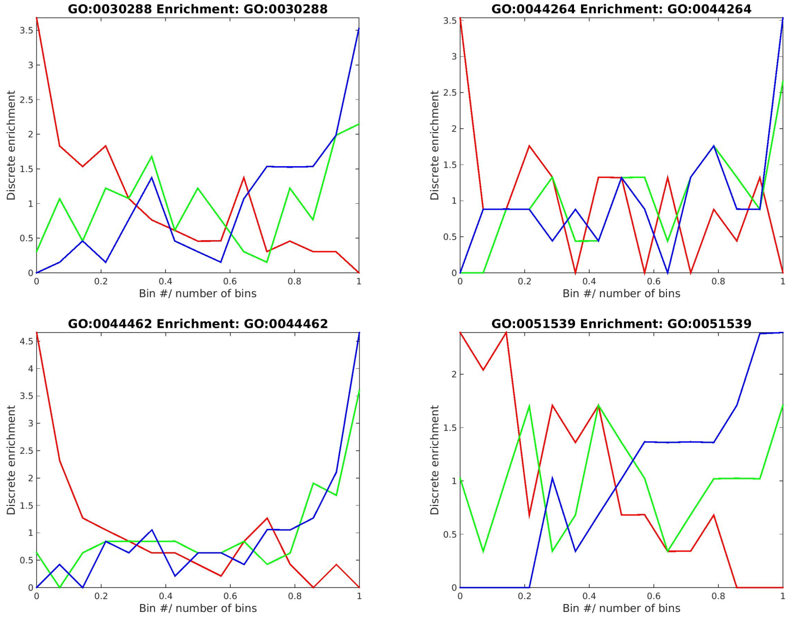

Figure 12: Left Vertex Density enrichments are shown in the case of 15 bins and FDR<0.05.

1.4.3 Archetype 3

As seen in the section above, cytoplasmic proteins, which are characterized by high solubility and low hydrophobicity, cluster at the top vertex. These proteins are specialized in *regulation processes*, as derived from the enrichment analysis of the GO terms. In the figure 9 below we have examples of enriched regulation processes, such as: regulation of biological processes (GO:0050789), regulation of metabolic processes (GO:0019222), biological regulation (GO:0065007) and regulation of primary metabolic processes (GO:0080090). The cytoplasmic characteristic of these proteins is supported also by the cellular component *cytosol component* (GO:0044445).

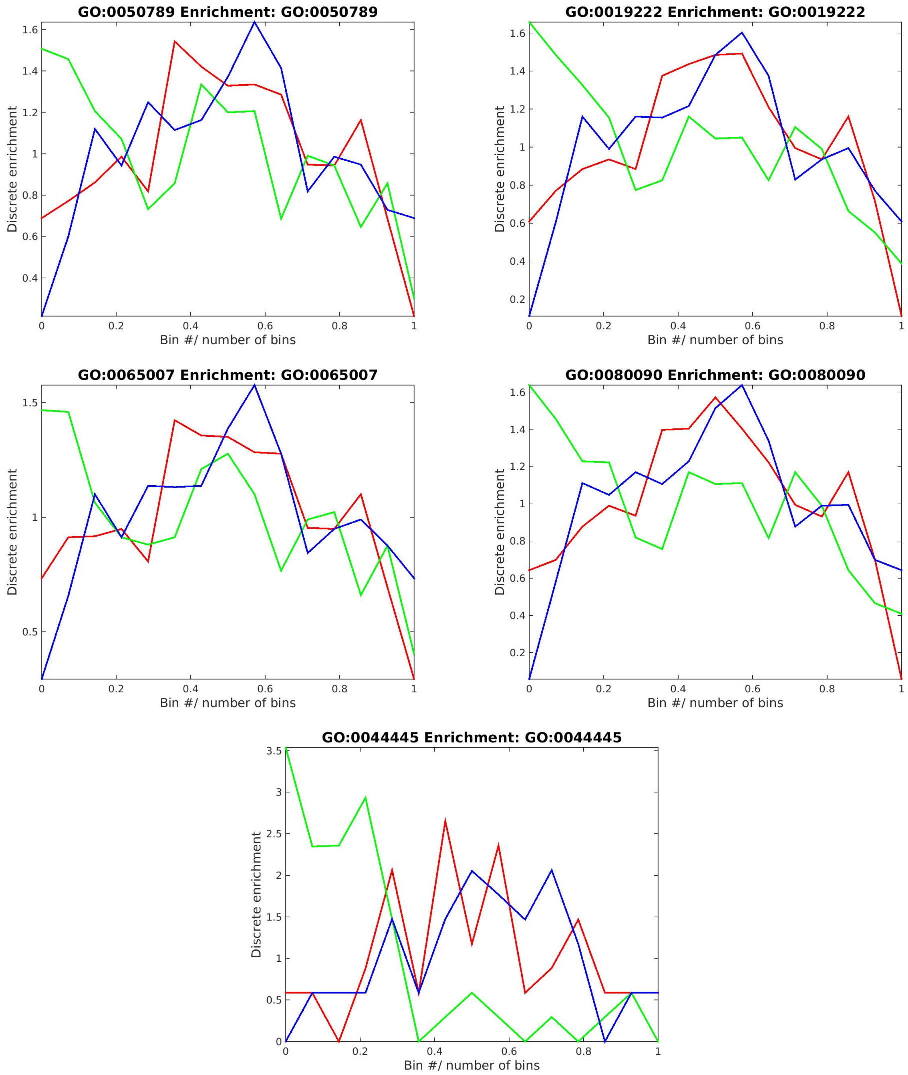

Figure 13: Top Vertex Density enrichments are shown in the case of 15 bins and FDR<0.05.

We end this section by introducing three generic GO-labels (see also figure 2 in the main text), which are useful to group the archetypal GO-annotations with each other in

three main classes. Enrichment analysis performed on this new labels can be considered as an average analysis of the archetypal annotations.

GO-annotations associated to Archetype 1, (GO:0005886, GO:0008324, GO:0006811, GO:0022804, GO:0034220, GO:0015075, GO:0015711, GO:0022891, GO:0031226, GO:0044765, GO:1902578), are thus relabelled as ”transportation”, those associated to Archetype 2 (the red one), (GO:0003674, GO:0005976, GO:0009057, GO:0016787, GO:0019867, GO:0030288, GO:0044264, GO:0044462, GO:0051539), are relabelled as ”porin-binding-polyssaccharyde”, while those associated to Archetype 3 (the green one), (GO:0050789, GO:0019222, GO:0044445, GO:0065007, GO:0080090), are thus relabelled as ”regulation”. In the Figures 14 and 15 below, we plot the displacement of the proteins pertaining to the three classes:

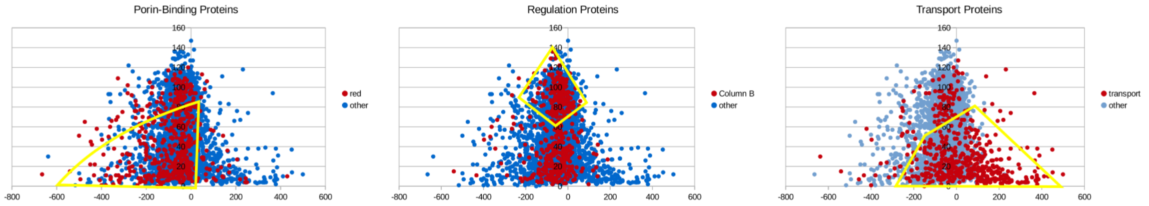

Figure 14: Density of the archetypal feature Proteins labelled with regulation proteins, porin-binding-polyssaccharyde, transport proteins are plotted in the space of solubility vs hydrophobicity. We enclose with a yellow convex hull the specialized proteins.

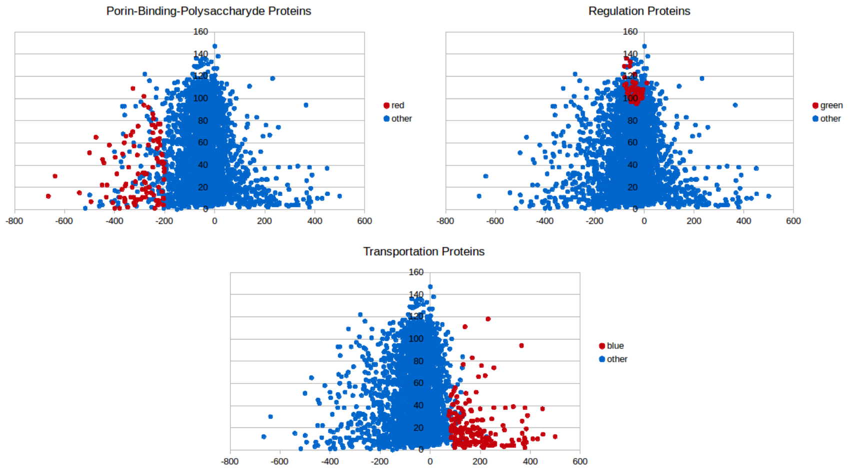

Figure 15: Archetypal proteins in the 1st bin. Red points denote the proteins with the given feature in the bin nearest each vertex (≈ 200 proteins).

Enrichment analysis performed on the three archetypal groups is shown below in the figure 16:

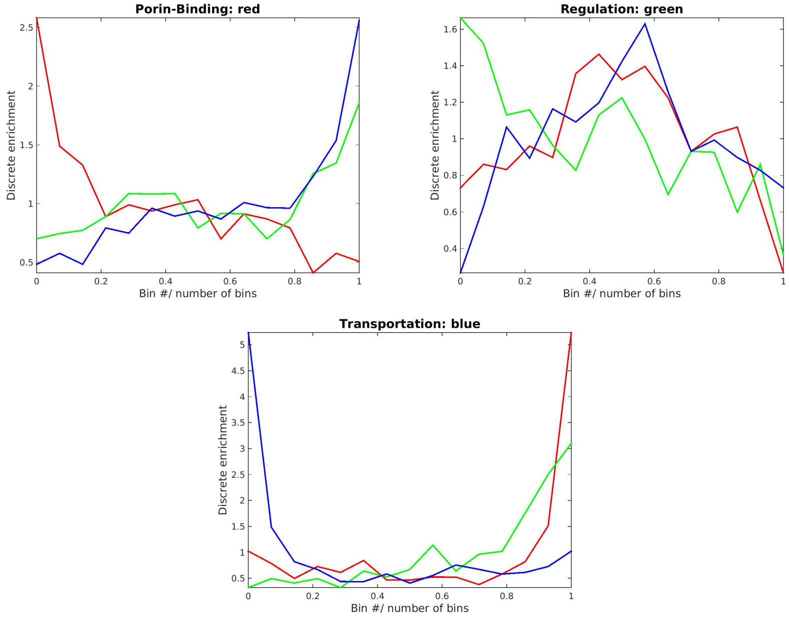

Figure 16: Enrichment analysis of the three main groups We binned the dataset into 15 bins. In panel a) porin-binding-polyssaccharyde proteins, b) regulation proteins, c) transportation proteins.

Statistical fluctuations increase with the number of bins. In the case of 25 bins the three archetypal groups have the following enrichment patterns:

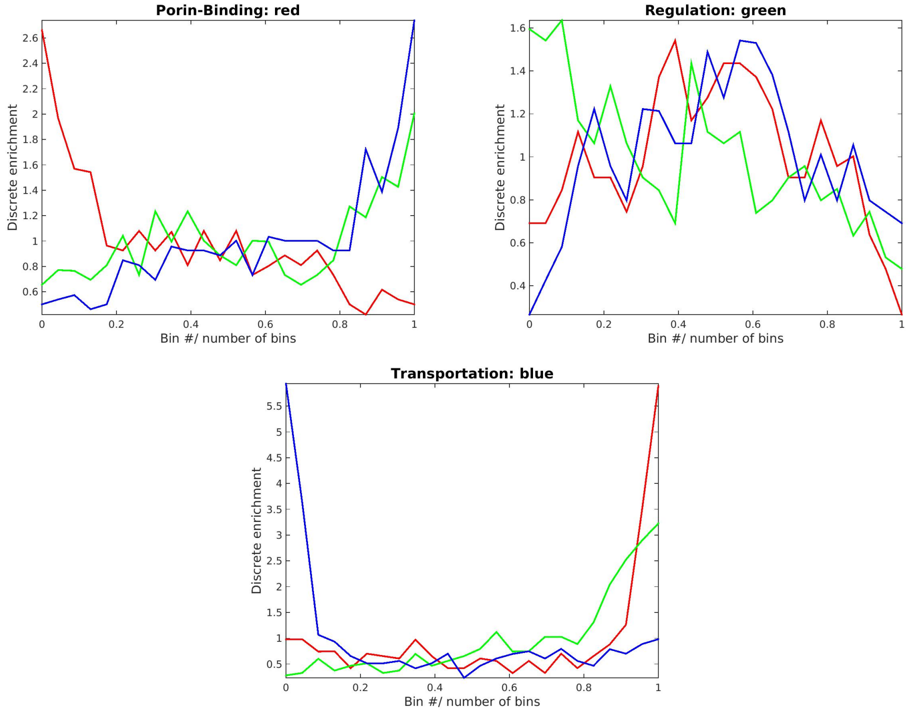

Figure 17: Enrichment analysis of the three main groups We binned the dataset into 25 bins.

2 Evidence for a Tetrahedron

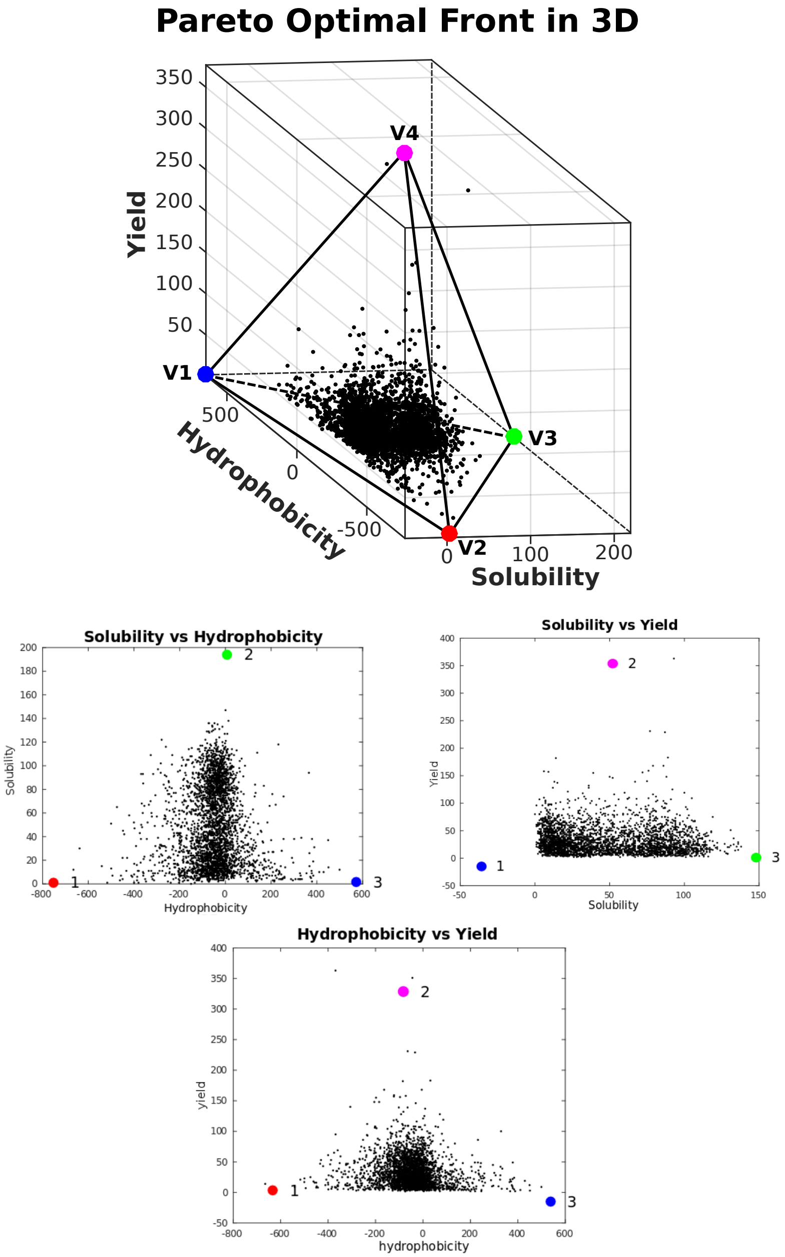

Figure 18: Tetrahedron projections Tetrahedron in the hydrophobicity-solubility-yield space. The three vertices in the hydrophobicity-solubility plane, correspond to the archetypes identified in the previous section.

Arch (Orig) Position	Hydrophobicity	Solubility	Yield
Red	-755.26	-2.62	5.57
Purple	-128.65	48.15	378.9
Green	13.31	211.37	-0.09
Blue	636.04	-34.12	1.84

Arch (PCA) Position	Hydrophobicity (PC1)	Solubility (PC2)	Yield (PC3)
Red	-703.60	-47.41	-53.32
Purple	-87.59	-49.51	340.54
Green	63.94	165.46	-7.68
Blue	687.40	-77.69	-23.15

Table 2: Coordinates of the four archetypes as found with Sisal. The coordinates of the four vertices in the solubility-hydrophobicity-yield space are shown in the top table, whereas the coordinates in the principal component space are shown in the bottom table.

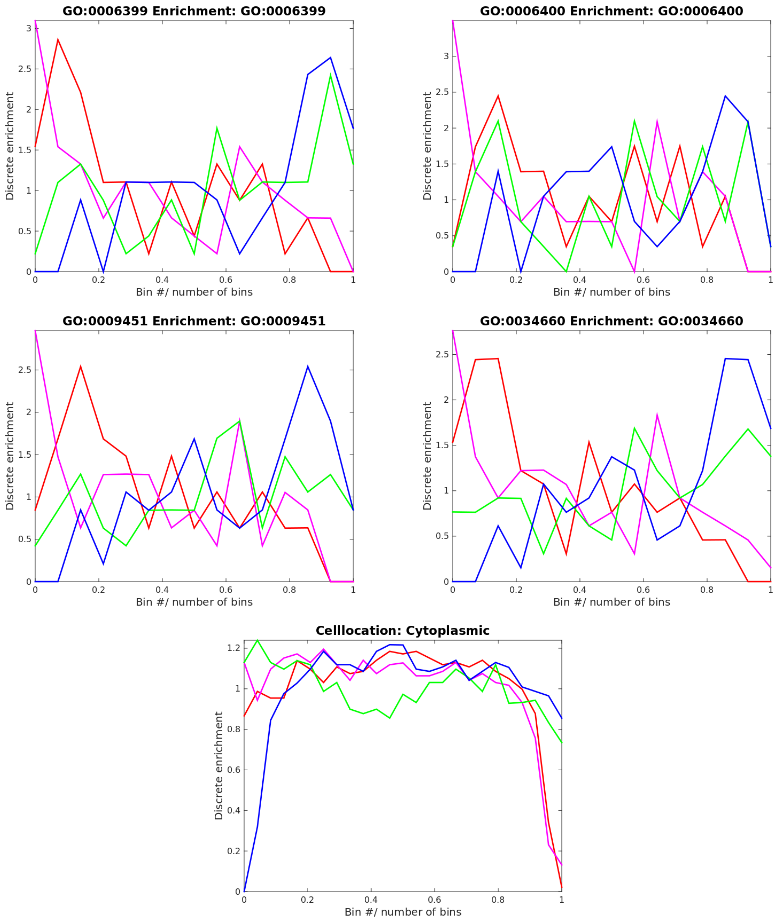

Figure 19: Fourth Vertex enrichments Density enrichments are shown in the case of 15 bins and FDR<0.05. We show the subcellular location in the case of 25 bins.

References

1. Mørup, M. Hansen L. K. Archetypal analysis for machine learning and data mining. Neurocomputing 80, 54-63 (2012).

2. Bioucas-Dias, J.M. in Hyperspectral Image Signal Process. Evol. Remote Sens. First Workshop 14 (IEEE, 2009).

3. Ashburner et al. Gene ontology: tool for the unification of biology (2000) Nat Genet 25(1):25-9

4. Hart, Y. Sheftel, H. Hausser, J. Szekely, P. Ben-Moshe, N.B. Korem, Y., Tendler, A. Mayo, A.E. Alon, U. Inferring biological tasks using Pareto analysis of high-dimensional data. Nature Methods 12, 233-235 (2015).

5. Benjamini, Y. Hochberg, Y. Controlling the false discovery rate: a practical and powerful approach to multiple testing. Journal of the royal statistical society. Series B (Methodological) 57, 289-300 (1995).

6. eSOL database(http://tp-esol.genes.nig.ac.jp/) developed in the Targeted Proteins Research Project.

7. Orfanoudaki, G. Economou, A. Proteome-wide subcellular topologies of E. coli polypeptides database (STEPdb). Molecular and Cellular Proteomics 13, 3674-3687 (2014).
